# Cytokine-Mediated Regulation of Plasma Cell Generation: IL-21 Takes Center Stage

**DOI:** 10.3389/fimmu.2014.00065

**Published:** 2014-02-18

**Authors:** Leen Moens, Stuart G. Tangye

**Affiliations:** ^1^Immunology and Immunodeficiency Group, Immunology Research Program, Garvan Institute of Medical Research, Darlinghurst, NSW, Australia; ^2^St Vincent’s Clinical School, University of New South Wales, Darlinghurst, NSW, Australia

**Keywords:** human B cells, differentiation, plasma cells, cytokines, IL-21, immunodeficiency, autoimmune diseases

## Abstract

During our life, we are surrounded by continuous threats from a diverse range of invading pathogens. Our immune system has evolved multiple mechanisms to efficiently deal with these threats so as to prevent them from causing disease. Terminal differentiation of mature B cells into plasma cells (PC) – the antibody (Ab) secreting cells of the immune system – is critical for the generation of protective and long-lived humoral immune responses. Indeed, efficient production of antigen (Ag)-specific Ab by activated B cells underlies the success of most currently available vaccines. The mature B-cell pool is composed of several subsets, distinguished from one according to size, surface marker expression, location, and Ag exposure, and they all have the capacity to differentiate into PCs. For a B-cell to acquire the capacity to produce Abs, it must undergo an extensive differentiation process driven by changes in gene expression. Two broad categories of Ags exist that cause B-cell activation and differentiation: T cell dependent (TD) or T cell independent (TI). In addition to the B-cell subset and nature of the Ag, it is important to consider the cytokine environment that can also influence how B-cell differentiation is achieved. Thus, while many cytokines can induce Ab-secretion by B cells after activation with mimics of TD and TI stimuli *in vitro*, they can have different efficacies and specificities, and can often preferentially induce production of one particular Ig isotype over another. Here, we will provide an overview of *in vitro* studies (mouse and human origin) that evaluated the role of different cytokines in inducing the differentiation of distinct B-cell subsets to the PC lineage. We will place particular emphasis on IL-21, which has emerged as the most potent inducer of terminal B-cell differentiation in humans. We will also focus on the role of IL-21 and defects in B-cell function and how these contribute to human immunopathologies such as primary immunodeficiencies and B-cell mediated autoimmune conditions.

## Introduction

The humoral arm of the immune system is critical for providing protective antibodies (Abs) against infection pathogens. The Ab pool is maintained by long-lived plasma cells (PCs), which continuously secrete Abs following their formation in response to exposure to specific antigen (Ag). In 1948, Fagraeus was the first to report that PCs are the outcome of terminal B-cell differentiation and demonstrated their importance to Ab production *in vitro* (1). We now know that B cells are capable of secreting multiple Ig isotypes (IgM, IgG, IgA, IgE) and subclasses of these isotypes (IgG_1–4_, IgA_1–2_) following the receipt of appropriate stimulate. However, today – 65 years later – our understanding of the complexities of PC development remains incomplete.

## Plasma Cell Formation: The Importance of T Cells, Cytokines, and Transcription Factors

**Figure 1:**
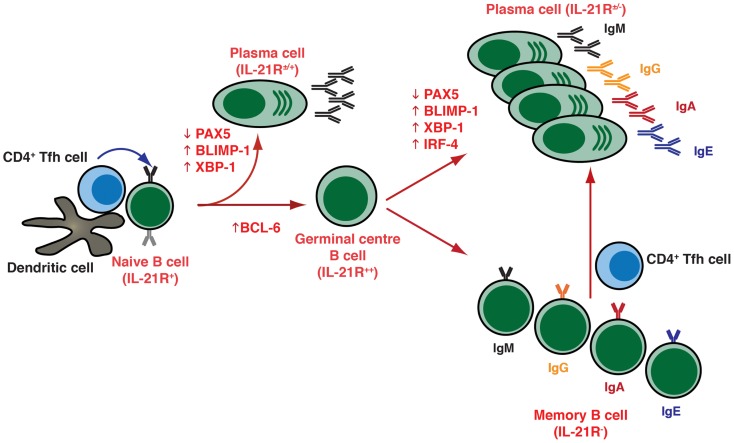
**T cell dependent B-cell differentiation**. Following the receipt of signals provided by the microenvironment [e.g., Ag, CD4^+^ T (Tfh) cells, DC], naïve B cells undergo activation and can initially differentiate into either extrafollicular short-lived Ab-secreting plasma cells (secreting predominantly IgM), or can seed a germinal center (GC). Within GCs, B cells undergo somatic hypermutation of their Ig V region genes and only those B cells with the highest affinity are selected to then differentiate into long-lived memory B cells or plasma cells that are capable of secreting a variety of Ig isotypes, including the switched isotypes IgG, IgA, and IgE. The outcome of the GC reaction is heavily influenced by Tfh cells, especially those within the GC itself. These cells are not depicted on the figure but they contribute greatly at this stage of B-cell differentiation. Following re-encounter with the initiating Ag, memory B cells rapidly differentiate into plasma cells. The differentiation of naïve B cells to these distinct effector fates is controlled by the balanced expression and regulated function of various transcription factors, including (but not exclusively) PAX5, BCL-6, BLIMP-1, XBP-1, and IRF4.

Plasma cells are generated as a result of cognate interactions between Ag-specific B cells, CD4^+^ T helper cells, and dendritic cells in response to foreign Ags (Figure 1). These interactions can drive B cells to become low-affinity short-lived, predominantly IgM-secreting, plasmablasts that provide an initial wave of protection against invading pathogens. More importantly though, they also lead to the formation of germinal centers (GCs), which are specialized structures in the follicles of secondary lymphoid tissues where somatic hypermutation (SHM) of immunoglobulin (Ig) variable region genes and selection of high-affinity B cells occurs. These selected high-affinity variants can then differentiate into long-lived memory B cells or PCs (2, 3) (Figure 1). This differentiation event is in part mediated by T follicular helper (Tfh) cells, a distinct subset of CD4^+^ T cells characterized by expression of the transcriptional repressor B-cell lymphoma-6 (Bcl-6), the surface markers CXCR5, PD-1, ICOS, and CD40 ligand (CD40L), and production of various cytokines including interleukin-4 (IL-4), IL-10, and IL-21. Tfh cells localize to follicles and GCs – where they are termed “GC Tfh cells” – where they can interact with B cells and instruct their maturation into memory cells or PCs (4–6).

The differentiation of activated B cells into PC is regulated by transcriptional programs and networks that are influenced by numerous inputs and microenvironmental factors. These include the nature of the Ag and of the responding B-cell subset, the location in which Ag encounter occurs, and the accessory cells involved (7, 8). The key transcription factors involved in regulating PC formation include the transcriptional repressors Bcl-6 and B-lymphocyte induced maturation protein (BLIMP)-1, encoded by the PRDM1 gene, as well as transcription factors PAX5, X-box-binding protein-1 (XBP-1), and IFN-induced regulatory factor 4 (IRF4) (Figure 1) (7, 8). Thus, while Bcl-6 is expressed in GC B cells and is required for the GC formation (9–11), it blocks PC differentiation and maintains a GC B-cell fate by suppressing expression of BLIMP-1, which is considered the master regulator of PC differentiation, being required for – or at least correlated with – PC commitment in mice and humans (Figure 1) (12–15). BLIMP-1 expression controls PC differentiation by restraining the mature B-cell gene expression program by down-regulating a set of genes including MHC, CIITA, PAX5, and CMYC, which result in a decrease of MHC class II expression, loss of B-cell identity, and cessation of proliferation, respectively (8, 14). BLIMP-1 may also co-ordinate expression of XBP 1, which allows expansion of the secretory apparatus necessary for high-level protein synthesis in PC differentiation (Figure 1) (16).

Cytokines represent a diverse group of small soluble proteins that can function as growth and differentiation factors in autocrine or paracrine ways. Cytokines exhibit considerable redundancy, in that many cytokines share similar functions. Through binding to specific cell surface receptors, they initiate signal transduction pathways that are critical for a diverse spectrum of functions, including induction of immune responses, cell proliferation, differentiation, and apoptosis. The key contribution of cytokines to B-cell differentiation lies in their ability to modulate expression of these transcription factors such that they regulate Ig secretion by B cells activated with mimics of T cell dependent (TD) (e.g., CD40L) or T cell independent (TI) [e.g., engaging the B-cell receptor (BCR), Toll-like receptors (TLRs)] stimuli *in vitro* and, by extension, *in vivo*. The effects of cytokines on B-cell differentiation is evidenced not only by the magnitude of the Ab response but also the quality, in terms of the particular Ig isotype(s) induced. Although many cytokines are capable of promoting B-cell differentiation, the relative roles of specific factors, and the hierarchy of the interactions between several cytokines, has only emerged in the last 10 years.

## Discovery of T Cell-Derived Factors as Critical Mediators of B-Cell Differentiation and PC Generation

The concept that cross-linking of the BCR initiates B-cell activation and facilitates these cells to respond to T-cell-derived soluble factors and undergo proliferation and differentiation to become Ab-secreting cells was first appreciated in the 1970s (17–20). The different factors were classically grouped as T cell-replacing factors, some of which influence the replication of B cells (B-cell growth factor), while others directly cause B-cell differentiation to Ab-secretion cells (B-cell differentiation factor) (21). While it gradually emerged that these T cell-derived factors are Ag non-specific, genetically non-restricted, and are indeed involved in the differentiation of B cells into Ab-secreting cells, at this time no single factor had been isolated or molecularly cloned, and it remained unknown how many factors were actually involved in, or required for, B-cell terminal differentiation (22, 23).

**Figure 2:**
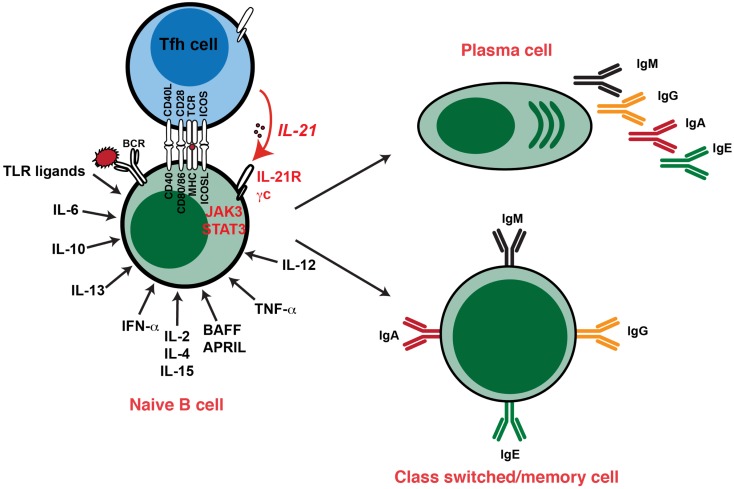
**Cytokine-induced differentiation of human B cells *in vitro*: requirement for IL-21 signaling *in vivo***. *In vitro* studies demonstrated that human B cells could undergo events such as Ig class switching and differentiation to become Ig-secreting cells following stimulation with a diverse range of cytokines. However, analysis of individuals with hypomorphic mutations in genes encoding STAT3, γc (*IL2RG*), JAK3, or IL-21R have revealed that this pathway – activated by IL-21 – is critical for the generation of memory B cells and the establishment of Ag-specific Abs *in vivo*. Thus, although cytokines such as IL-4, IL-13, IL-10, and BAFF/APRIL are strong B-cell growth and differentiation factors, their function is insufficient to compensate for impaired IL-21/IL-21R signaling *in vivo* in the setting of generating robust, long-lived Ag-specific Ab, and memory responses. Consequently, IL-21-mediated B-cell activation is a necessary and sufficient step in the generation of protective long-lived humoral immune responses in humans.

The molecular revolution of the 1980s saw the cloning and characterization of several cytokines – IL-2, IL-4, IL-5, IL-6, IFNs – which had B-cell growth and differentiation capacity (Table 1). This continued into the 1990s with the discovery of IL-10, IL-12, IL-13, IL-15, TNFα, BAFF, and APRIL, which could promote various aspects of B-cell function (Figure 2; Table 1). Thus, these cytokines enhanced proliferation and induced isotype switching, PC formation, and Ig secretion by activated B cells (22–47) (Table 1; Figure 2). Importantly, this era also saw the identification of CD40L – transiently expressed on the surface of activated CD4^+^ T cells – which, together with these cytokines, was revealed to be a critical regulator of many facets of B-cell biology (48). Specifically, while CD40L (or anti-CD40 mAb) itself had minimal effect on Ab-secretion by murine and human B cells, Ab-secretion could be induced in an isotype specific manner in the presence of exogenous cytokines (Figure 2; Table 1). Thus, IL-4 and IL-13 directs naïve human B cells to switch to IgG_4_ and IgE expression and production, while IL-4 exerts a similar effect for inducing IgG_1_ and IgE by murine B cells (Table 1), with IL-5 acting synergistically with IL-4 in these murine B-cell responses (25, 28, 29, 41, 49–51). The significance of these *in vitro* findings was underscored by the generation of IL-4 deficient mice, which had significantly reduced production of IgE following nematode infection (52). Interestingly, IL-4-induced IgE production by human B cells could be enhanced by IL-6 or TNFα (33, 45), or inhibited by IL-8 (53), IL-12 (54), or IFN-α or IFN-γ (33, 40). While murine B cells were initially reported to be unresponsive to IL-13 (55), subsequent studies noted that IL-13 could enhance Ab production by murine B cells *in vivo* and that it acts directly on B cells *in vitro* to increase survival, thereby increasing Ab production (56). Additional support for a role for IL-13 in modulating murine B cells came from the analysis of IL-13 transgenic mice, which exhibited substantially increased levels of serum IgE, even in the absence of IL-4 (57). Similarly, while deficiency of either IL-4 or IL-13 reduced the levels of Ag-specific IgE, combined deficiency of both IL-4 and IL-13 resulted in undetectable levels of IgE (58). Thus, it is likely that IL-4 and IL-13 co-operate in both mice and humans to regulate Ig class switching, especially to IgE. IL-10 also strongly modulated the behavior of human B cells, significantly increasing the levels of IgM, IgG_1_, and IgA secreted by human B cells stimulated through CD40 or the BCR (42). IL-10 was also found to induce class switching in human naïve B cells to IgG_1_ and IgG_3_ (59), and together with TGF-β promoted switching to IgA (31). IL-10 also mediated the differentiation of GC and memory B cells to PCs (Table 1) (26). The ability of IL-4, IL-10, and IL-13 to induce isotype switching reflected their abilities to upregulate expression of activation induced cytidine deaminase (AICDA), an enzyme critical for class switch recombination, while IL-10 mediated PC generation by inducing BLIMP-1 (7, 8, 60). The effects of IL-10, however, appear to be species specific because serum Ig levels were unaffected in mice that were either deficient for IL-10 or that expressed IL-10 from a transgene (61, 62). Similar to CD40L, the membrane bound form of TNF-α was also found to be transiently expressed on human activated CD4^+^ T cells, and could co-stimulate polyclonal Ig secretion induced in human B cells co-cultured with mitogen-stimulated CD4^+^ T cells, or their membranes, together with IL-4 (27, 63) (Table 1).

**Table 1:** **Contribution of different cytokines to the *in vitro* behavior of human B cells**.

Cytokine	Effect on B cells	Reference
CD40L	Induces activation, blastogenesis, proliferation	(25 )
IL-2	Enhances proliferation of CD40L-stimulated B cells	(25, 26, 30, 36, 38, 39, 44, 104, 144, 145 )
	Co-operates with other cytokines/stimulatory factors to enhance differentiation of activated B cells	
IL-4	Enhances proliferation induced by CD40L, BCR engagement	(25, 33, 37, 40, 45 )
	Induces expression of AICDA	
	Induces CSR, preferentially to IgG1, IgG4, and IgE	
IL-6	Promotes survival and function of *in vitro*-derived as well as primary and malignant plasma cells	(32, 44 –47 )
IL-10	Enhances proliferation induced by CD40L, BCR engagement	(25, 26, 31, 34, 42, 59, 60, 144 )
	Induces expression of AICDA, BLIMP-1	
	Induces CSR, preferentially to IgG1, IgG3	
	Co-operates with TGF-β to induce CSR to IgA	
	Promotes differentiation of B cells to become plasma cells secreting IgM, IgG, IgA	
IL-12	Induces B cells to differentiate into IgM-secreting cells	(32, 54 )
	Co-operates with IL-6 to augment IgM secretion	
	Suppresses IL-4-induced IgE production	
IL-13	Enhances proliferation induced by CD40L, BCR engagement	(28, 29, 41, 50 )
	Induces expression of AICDA	
	Induces CSR, preferentially to IgG1, IgG4, and IgE	
	Effects essentially overlap with those of IL-4	
IL-15	Enhances proliferation of B cells stimulated with CD40L or BCR engagement	(24 )
	Induces secretion of IgM, IgG1, and IgA by CD40L-stimulated B cells	
	Magnitude of the effect was comparable to IL-2	
IL-21	Currently, the most potent cytokine identified capable of regulating human B-cell function	(60, 71, 76, 83, 84, 96 –101, 103, 106, 107, 121 )
	Enhances proliferation induced by CD40L, BCR engagement	
	Induces expression of AICDA, BCL-6, BLIMP-1, XBP-1	
	Induces CSR, preferentially to IgG1, IgG3, and IgA1	
	Promotes differentiation of B cells to become plasma cells secreting IgM, IgG, IgA, and IgE	
	Synergizes with IL-4 for CSR to IgG and secretion of IgE	
	Sustain survival of primary plasma cells present in secondary lymphoid organs	
	Growth and survival factor for malignant plasma cells (i.e., myeloma)	
	Requires functional STAT3 to induce plasma cells differentiation	
IFNα, IFNγ	Inhibits CD40L-induced B-cell proliferation	(25, 33, 36, 40 )
	Inhibits IL-4 induced IgE secretion	
	IFNα primes activated B cells to differentiate into precursors of plasmablasts, that become plasmablasts in response to IL-6	
TNFα	Membrane TNFα expressed by CD4+ T cells acts as a co-stimulus to promote B-cell differentiation induced by CD40L and IL-4	(27, 33, 63)
BAFF/APRIL	BAFF promotes survival of transitional B cells, as well as of early plasma cells and some malignant plasma cells	(64 –66 )
	BAFF and TACI can induce CSR to various isotypes, and can induce secretion of these Ig’s when combined with BCR signaling and cytokines (e.g., IL-4, IL-10, IL-15)	
TGFβ	Inhibits IL-4 induced IgE secretion	(31, 33 )
	Can induce CSR to IgA, in combination with IL-10	

*CSR, class switch recombination*.

IL-2 has had a long history of being documented of enhancing Ig secretion by activated human B cells (Table 1) (38, 39). Consistent with the structural and functional similarities between IL-2 and IL-15, it was not surprising that IL-15 could also stimulate proliferation and induce secretion of IgM, IgG_1_, and IgA, but not IgG_4_ or IgE, by CD40L-primed B cells. This activity of IL-15 was comparable to that of IL-2 (24).

More recently, the TNF-related molecule BAFF, and its homolog APRIL, has emerged as a global regulator of B-cell development and function (64–66). While a primary role for BAFF lies in the ability to promote the survival of B cells at the transitional stage of development (65), both BAFF and APRIL can also induce the molecular events associated with isotype switching to IgG and IgA, and to IgE in the presence of IL-4. Furthermore, the secretion of these Ig isotypes occurred when the B cells also received signals through the BCR (64, 66). BAFF and APRIL can also sustain the survival of PCs *in vivo* and *in vitro* (66, 67). BAFF functions by binding to the surface receptors BAFF-R, TACI, or BCMA; APRIL can also activate B cells by binding to TACI and BCMA (65, 66) (Table 1). Interestingly, these effects of BAFF and APRIL appear to be mediated through different receptors. Thus, the pro-survival effects of BAFF on transitional and naïve B cells are delivered through BAFF-R, while this effect on PCs occurs predominantly through BCMA. On the other hand, BAFF-R and TACI mediates isotype switching to IgG, IgA, and IgE induced by BAFF and APRIL, respectively (64–66). Lastly, heparan sulfate proteoglycans can also act as a receptor for APRIL, and this appears to be important for mediating the pro-survival effects of APRIL on BM PCs (67–69).

Collectively, it is clear that myriad cytokines and combinations thereof, are capable of eliciting activation and terminal differentiation of human B cells to differing extents. However, with the discover of IL-21 in 2001, and the subsequent characterization of its function on human and murine B cells during the following decade, the physiological significance of many of these factors in initiating humoral immune responses needs to be re-addressed as IL-21 has emerged as the most potent inducer of B cell differentiation.

## Pleiotropic Effects of IL-21 on Human and Murine B-Cell Differentiation

IL-21 belongs to the type I family of cytokines that also includes IL-2, IL-4, IL-7, IL-9, and IL-15, all of which bind to and form a complex with the common γ-chain (γc) and their private receptors (70–73). The IL-21 receptor (IL-21R) is expressed by fibroblasts, keratinocytes, and intestinal epithelial cells, but more importantly is also expressed on lymphocytes (T, B, NK cells), macrophages, and dendritic cells, and the levels of expression can be increased following cellular activation (70, 71, 74–77). IL-21 is predominantly produced by activated CD4^+^ T cells and NKT cells (78–80), with the greatest production being by Tfh and GC Tfh cells (4–6). Akin to most cytokines, IL-21 exerts its effect by activating Janus kinase/signal transducers and activators of transcription (JAK/STAT) signaling pathways, specifically Jak1 and Jak3, and STAT1, STAT3, and to a lesser extent STAT5 (72, 81–84). The initial description of IL-21 hinted at its B-cell tropism, inasmuch that Parrish-Novak et al. showed that IL-21 significantly co-stimulated proliferation of human blood B cells induced by anti-CD40 mAbs (71). Since then, several studies have confirmed that IL-21 is an important regulator of B-cell activation, proliferation, PC differentiation, and Ab-secretion in both mice and humans.

### The role of IL-21 in murine activated B-cell proliferation, apoptosis, PC differentiation, Ab-secretion, and memory B-cell formation

In a seminal study, Ozaki et al. demonstrated that the IL-21 signaling pathway is involved in regulating Ab production and isotype switching (85). They showed that IL-21R^−/−^ mice, despite having normal lymphoid development, have significantly diminished total serum and Ag-specific IgG_1_ titers but elevated IgE levels in response to TD Ag immunization compared to wild-type animals. Ag-specific IgG_2b_ and IgG_3_ serum levels were also decreased whereas IgG_2a_ and IgM titers were largely unaffected in the absence of the IL-21R. The decreased IgG_1_ response appeared to result from a reduction in the generation of Ag-specific IgG_1_ producing PCs. These *in vivo* data established that IL-21 has a critical role in inducing IgG_1_ production, while concomitantly suppressing IgE responses. Strikingly, IL-4^−/−^IL-21R^−/−^ double-knockout mice displayed a more severe phenotype, characterized by a more dramatically reduced IgG response. Furthermore, the strong up-regulation of IgE secretion in IL-21R^−/−^ mice was abrogated in IL-4^−/−^IL-21R^−/−^ mice indicating that the “hyper-IgE” phenotype of IL-21R^−/−^ mice was dependent on IL-4 (85). Importantly, these *in vivo* findings were complemented by *in vitro* investigation of the effects of IL-21 on murine B cells. Thus, IL-21 enhanced proliferation of anti-IgM and/or anti-CD40 mAb-stimulated murine B cells and initiated PC differentiation and class switching, as revealed by increased expression of Syndecan-1 (CD138) and surface IgG1 on these cells (86).

These findings provided strong evidence that IL-21 is likely to achieve its potent effect on humoral immune responses *in vivo* by acting directly on B cells. Indeed, this has been verified in a series of studies where IL-21R-sufficient or deficient B cells were adoptively transferred into recipient mice, and the B-cell response to TD Ags or pathogens then tracked. It was generally found that when B cells were unable to respond to IL-21, humoral immunity was compromised with impaired formation of GC, with respect to magnitude and/or kinetics, and of long-lived Ag-specific PC. The mechanism underlying aberrant GC formation was suboptimal induction of Bcl-6 expression in GC B cells, which attenuated affinity maturation and selection of high-affinity variants. Although memory cells were generated in normal numbers from IL-21R-deficient B cells, the IL-21R-deficient memory cells were unable to respond to secondary challenge with specific Ag, resulting in ineffective recall responses. In contrast to the GC response, the generation of extrafollicular plasmablasts in response to pathogens was unaffected by B-cell specific IL-21R-deficiency (87– 92). IL-21 can activate STAT3 (72, 81– 84). Intriguingly, analysis of STAT3^flox/flox^ CD19^cre^ mice showed some similarities to mice whose B cells lacked IL-21R. Specifically, STAT3^flox/flox^ CD19^cre^ mice have normal levels of serum IgM, IgA, and IgG, but a large reduction in Ag-specific serum IgG_1_ levels and splenic PCs following immunization with TD Ags (93). This established that expression of STAT3 in B cells is important for TD differentiation of B cells into IgG_1_-secreting PC (93), with subsequent studies implicating IL-21 as being the key STAT3-activating cytokine potentially involved in this process (87–92). Thus, IL-21/IL-21R signaling, possibly via STAT3, in B cells appears to be required for the generation and maintenance of long-lived PC and humoral memory to TD Ags, but is dispensable for GC-independent Ab responses.

Given the importance of IL-21R expression for normal Ig production *in vivo* (85, 86), a surprising finding was that murine IL-21 could inhibit B-cell proliferation induced by either anti-IgM and IL-4, or TLR ligands such as LPS or CpG (94, 95). Furthermore, although IL-21 impressively promoted proliferation of CD40-activated B cells, the proportion of B cells that was apoptotic in the presence of IL-21 exceeded that observed in its absence (95). Induction of apoptosis by IL-21 in both resting and activated murine B cells correlated with reduced expression of Bcl-x_L_ and Bcl-2 and elevated expression of Bim (94, 95). Consistent with this, IL-21-induced apoptosis could be prevented by restoring expression of Bcl-x_L_ or Bcl-2 either by overexpressing these proteins or inducing their expression by activation prior to exposure to IL-21 (94). Increased B-cell apoptosis was also observed *in vivo* in mice either transgenic for IL-21 or that received IL-21 administered via hydrodynamic-based delivery of plasmid DNA. Thus, it appears that IL-21 can differential influence B-cell fate depending on the signaling context (86).

Together, these data show that IL-21 is an important factor for the activation, proliferation, differentiation, Ag production, or death of murine B cells, with the outcome being dependent on the context of co-stimulation. The defect in GC-dependent Ab production in IL-21/IL-21R deficient mice after immunization indicates that differentiation into PCs may be a non-redundant activity of IL-21.

### IL-21 and human B cells

**Figure 3:**
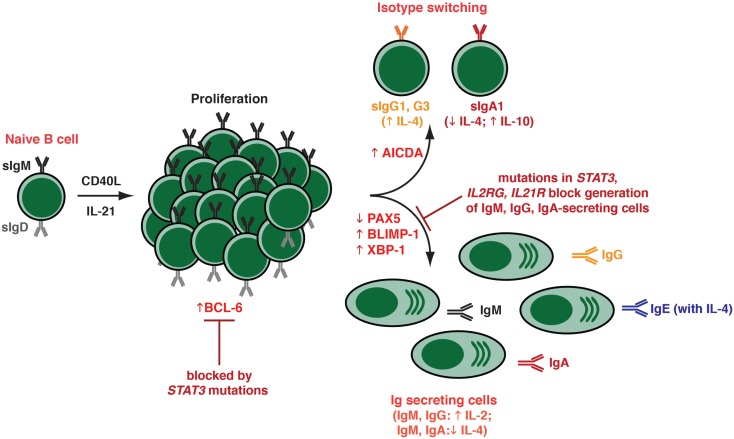
**Effects of IL-21 on human naïve B cells *in vitro***. When human naïve B cells are stimulated with CD40L together with IL-21, they were found to undergo intense proliferation. This was followed by induction of Ig class switching – predominantly to IgG_3_ and IgG_1_; a lesser extent to IgA_1_ – as determined by acquisition of expression of switched isotypes or differentiation to plasma-like cells capable of secreting all major Ig isotypes. The B cells that had undergone switching to become IgG^+^ or IgA^+^ were distinct from those that committed to a plasma cell fate – thus, the secreted Ig detected in these cultures was derived from the *in vitro*-derived plasma cells rather than the surface IgG^+^/IgA^+^ class switched cells. Both class switching and plasma cell formation induced by IL-21 could be modulated by additional cytokines, such as IL-4 (promoted switching to IgG; inhibited switching to IgA; increased secretion of IgG and IgE; suppressed secretion of IgM, IgA), IL-10 (increased IgA secretion), and IL-2 (increased secretion of IgM, IgG). These differentiation events coincided with the induction in expression of BCL-6, AICDA (required for class switching), and BLIMP-1/XBP-1 (required for plasma cell formation). The ability of IL-21 to induce naïve B cells to differentiate into plasmablasts/plasma cells *in vitro* was abolished by hypomorphic mutations in *STAT3*, as well as null mutations in *IL21R* or *IL2RG*.

Initial studies into the stimulatory effect of IL-21 revealed that IL-21 potently enhanced the proliferation of CD40-stimulated human B cells, with memory B cells undergoing a much stronger proliferative response than naïve B cells (Figure 3; Table 1) (96). Despite memory B cells proliferating more than naïve B cells in response to IL-21, the overall effect of IL-21 appeared to be greater on naïve than on memory cells. Thus, naïve B cells stimulated with CD40L/IL-21 exhibited a greater enhancement in their response, as well as a greater reduction in their time to enter cell division, over that induced by CD40L alone than did memory B cells (76). This is probably due to the basal expression of IL-21R on naïve B cells, whereas it is absent from memory cells (Figure 1). Although expression of IL-21R increases following activation on naïve and memory B cells, it remained higher on the naive subset (76).

In terms of differentiation, when total CD19^+^ splenic B cells were stimulated *in vitro* with anti-CD40 mAb in the presence of IL-21, they were induced to secrete IgM and IgG in an IL-21 dose-dependent manner (96). Pene et al. also made the important observation that IL-21 specifically induced production of IgG_1_ and IgG_3_ by human naïve B cells, demonstrating IL-21 to be a switch factor for these IgG subclasses (96). The findings from this elegant study were confirmed by several groups who also found that IL-21 induced proliferation as well as expression and secretion of IgM, IgG (predominantly IgG_3_) as well as IgA (mostly IgA_1_), and IgE by CD40L-stimulated naïve B cells that had been isolated from distinct anatomical sites, including umbilical cord blood, spleen, tonsils, and adult peripheral blood (Figure 3) (60, 76, 97–100) (Table 1). IL-21 also strongly induced Ig secretion from memory and GC B cells isolated from these sites (60, 97, 98, 100). The ability of IL-21 to induce such impressive Ig secretion correlated with the appearance of a substantial proportion of PCs – phenotypically identified as CD19^lo^IgD^−^CD38^hi^ or CD20^lo^CD38^hi^CD27^hi^ cells – in cultures of IL-21-stimulated B cells (Figure 3) (60, 98). Interestingly, a recent report also found IL-21 could support the survival of and Ig secretion by PCs in secondary lymphoid organs, but not those in the bone marrow (101). This is consistent with the differential expression of IL-21R on PCs from these diverse sites (76, 98, 102) (Figure 1), and suggests that IL-21 contributes to humoral immunity not only by inducing PC from naïve, memory, and GC B cells, but also promoting the survival and function of these cells in lymphoid tissues before they alter their requirements for survival within niches in bone marrow (67). The ability of IL-21 to sustain survival of normal PCs is reminiscent of the finding that IL-21 can promote growth and survival of malignant PC in multiple myeloma (103).

When compared to other cytokines that have been characterized as B-cell growth and differentiation factors, the effect of IL-21 was found to exceed that of IL-2, IL-4, IL-13, and IL-10 by up to 100-fold (60, 76, 97, 98). However, the actions of IL-21 could be complemented by these cytokines (Table 1; Figure 3). For example, IL-4 increased the frequency of IgG^+^ cells generated from, and the amount of IgG secreted by, naïve B-cell precursors that had been stimulated with IL-21 (Figure 3) (96, 97). Interestingly, while IL-21 favored the induction of IgG_3_^+^ B cells, the combination of IL-4 and IL-21 resulted in the preferential generation of IgG_1_^+^ switched B cells, which mirrored the effect of IL-4 alone but the magnitude of the response was greater. IL-4 and IL-21 were also capable of acting synergistically to induce 10- to 100-fold higher levels of IgE by CD40L-stimulated naïve B cells over that observed with either cytokine alone (Figure 3) (96, 99). In contrast, IL-4 abolished not only IL-21-induced IgM secretion but also switching to and secretion of IgA (97, 98); on the other hand, IgA secretion induced by IL-21 was augmented by IL-10 (97). Lastly, IL-2 could enhance PC differentiation induced by IL-21 (Figure 3) (98, 104). This was achieved by IL-21 inducing expression of CD25 – a component of the IL-2R – on activated B cells (104).

The physiological significance of these effects of IL-21 on human B cells has been born from experiments that assessed the relative contribution(s) of CD4^+^ T cell-derived cytokines to TD B-cell differentiation *in vitro*. Using an *in vitro* system whereby human activated CD4^+^ T cells can induce Ig production by co-cultured B cells (105), several groups have established that neutralization of IL-21 significantly inhibited T cell-induced B-cell activation, proliferation, differentiation, Ig secretion, and PC survival (60, 101, 106). Delayed blockade of IL-21 also inhibited PC differentiation after initial B-cell expansion, indicating that IL-21 is required for B-cell proliferation and PC differentiation (106). The findings that IL-21 is highly expressed by Tfh cells (78), and the IL-21R is upregulated on GC B cells (76) is consistent with a model of Tfh cells interacting with GC B cells to induce their differentiation to memory cells and PC predominantly via the production and delivery of IL-21 (4–6).

## Mechanism of Action of IL-21

The ability of IL-21 to guide multiple fates in activated B cells – class switching to express downstream Ig isotypes, commitment to the PC lineage, as well as formation of GCs and memory B cells – reflects the ability of IL-21 to induce the molecular machinery required for these processes. Thus, IL-21 is capable of inducing expression of AICDA, BLIMP1/PRDM1, and XBP-1, as well as reducing expression of PAX5, in both human and murine B cells (Figure 3) (60, 83, 84, 86, 98). Collectively, these factors regulate class switching and PC formation (8). Interestingly, the ability of IL-4 to suppress the stimulatory effects of IL-21 on naïve B cells correlated with a reduction in BLIMP-1 expression (60). IL-21 could also induce BCL-6 (83, 86, 89, 92, 98), which would contribute to GC formation *in vivo* (Figure 3) (8). Thus, in the setting of TD B-cell activation, Tfh-derived IL-21 can induce B cells to express all of the machinery required to undergo the major fates of differentiation: GC B cells by induction of Bcl-6; PCs following induction of BLIMP-1, and class switched B cells by inducing AICDA. It is likely that IL-21 induces expression of these opposing transcriptional regulators (i.e., BLIMP-1, Bcl-6) in distinct subsets of B cells that will ultimately develop into either PC or memory B cells. However, these outputs will ultimately reflect the balance of signals received and integrated by the B cells, with the effect of IL-21 being influenced by inputs delivered via receptors including the BCR, other complimentary cytokine, and co-stimulatory receptors.

As IL-21 can activate several STATs (73), the relative contribution of individual STAT molecules has been assessed. Diehl et al. demonstrated that constitutive activation of STAT3 in primary human B cells induced BLIMP-1 expression and initiated B cell differentiation, yielding cells with a phenotype (CD38^high^CD20^−^CD19^low^HLA-DR^low^CD138^+^) consistent with PC as well as enhanced Ab-secretion (84). Importantly, up-regulation of BLIMP-1 alone was not sufficient for differentiation of primary human B cells into PCs; this event also required concomitant down-regulation of BCL-6 (84). This study was the first to propose that STAT3 was the predominant mediator of the differentiation effects that IL-21 has on human B cells. These were largely confirmed by the demonstration that induction of PRDM1, XBP-1, and BCL-6 by IL-21 were abolished in naïve B cells isolated from individuals with hypomorphic mutations in *STAT3*, while these responses were unaffected by loss-of-function mutations in *STAT1* (83, 107). Intriguingly, IL-21-induced expression of *AICDA* in naïve B cells, as well as of *PRDM1* and *XBP1* in memory B cells, still occurred despite the presence of hypomorphic *STAT3* mutations, suggesting that class switching in naïve B cells and PC differentiation from memory B cells requires less STAT3 function than does the generation of PC from naïve B cells (83, 107).

Interestingly, high-affinity signaling through the BCR on immortalized B-cell lines can activate STAT3 (108). Similarly, CD40L enhanced the expression of BLIMP-1 induced by IL-21/STAT3 signaling in a GC B cell-like human cell line, thereby maximizing PC differentiation (109). Thus, it is possible that signals integrated in B cells through receptors such as CD40 and the BCR can amplify the effects of IL-21 by modulating activating or function of STAT3. It is also worth noting that STAT3 activation is important for the survival of multiple myeloma cells (110). As IL-21 is also anti-apoptotic for myeloma cells, it is tempting to speculate that IL-21 could contribute to STAT3 activation *in vivo* in the setting of this malignancy. Collectively, these studies have illuminated the pivotal role of IL-21-mediated STAT3 signaling in guiding key events of human B-cell differentiation.

## Lessons from Primary Immunodeficiencies

Primary immunodeficiencies (PIDs) result from monogenic mutations that compromise the ability of affected individuals to elicit appropriate immune responses. Consequently, these individuals exhibit susceptibility to infectious diseases and are often unable to respond to vaccination. As the genetic lesion is known in many PIDs, these conditions can reveal the unique functions of specific genes and related signaling pathways in immune cells and the importance of these pathways in productive and protective immune responses. Thus, analysis of PIDs can shed new light on the requirements for lymphocyte development and function. Indeed, several PIDs have confirmed the critical role played by IL-21 in humoral immunity in humans.

Heterozygous mutations in *STAT3* are the major cause of autosomal dominant hyper-IgE syndrome (AD-HIES) (111, 112), a multisystem disease affecting the immune and musculoskeletal systems (113, 114). Immunological defects include skin lesions, recurrent mucocutaneous invasive infections with *S. aureus* and *Candida*. These patients have normal serum levels of IgM, IgG, and IgA but increased levels of IgE (113, 114). Although the frequencies of total peripheral blood B cells are not significantly different between AD-HIES patients and control individuals, STAT3 deficiency impaired the *in vivo* generation of human memory B cells as well as the generation of Ag-specific Ab-secreting B cells and high-affinity serum Abs (Figure 2) (83, 107). This reduced number of memory B cells is in line with previously reported defective functional Ab responses in AD-HIES patients (115–118). Cytokines known to be involved in human B cell differentiation are IL-6, IL-10, and IL-21. Consistent with reduced memory B cells and poor induction of Ag-specific Ab responses in AD-HIES, naïve B cells from these patients were unable to respond to the stimulatory effects of IL-10 or IL-21 with respect to differentiation into PC *in vitro* (Figure 3). *STAT3* mutations also compromised the ability of IL-21 to prime B cells to the stimulatory effects of IL-2, inasmuch that induction of CD25 – and subsequent responsiveness to IL-2 – was attenuated on IL-21-stimulated STAT3-deficient human naïve B cells (104). These findings revealed that STAT3 plays a non-redundant role in generating Ag-specific memory B cells and Ab-secreting cells *in vivo*. However, it remained to be determined which STAT3-activating cytokine was requisite for these effects. This became clearer by examining patients with mutations in *IL2RG*, encoding γc, or *JAK3*, which associates with γc and delivers signals downstream of γc-containing cytokine receptors (73), that cause X-linked severe combined immunodeficiency (X-SCID) or one type of autosomal recessive (AR) SCID, respectively (73, 119). These PIDs are fatal unless treated by hematopoietic stem cell transplant (HSCT) (119).

X-linked severe combined immunodeficiency and JAK3 deficiency are characterized by a lack of T and NK cells but normal or increased numbers of B cells. However, due to the lack of CD4^+^ T cell help, B cell responses are impaired (119). While HSCT corrects the humoral defect in ~50% of patients, the remainder still requires ongoing Ig replacement therapy (120). One of the explanations for this is split chimerism, where donor-derived T cells successfully engraft in the recipient, but autologous host-derived B cells persist (120). Thus, despite the presence of functional CD4^+^ T cells, the *IL2G*/*JAK3* mutant B cells remain unable to respond to T-cell-derived helper signals, rendering the patient immunodeficient with respect to humoral immune responses (119, 120). We took advantage of this chimeric state to examine the B-cell compartment of X-SCID and JAK3 deficient patients who had undergone HSCT (121). Although *IL-2RG*/*JAK3* mutant naïve B cells responded normally to co-stimulatory signals delivered through the BCR, TLRs, and receptors for IL-10, IL-13, and even IL-4 [which can also signal through the IL13R; (73)], these B cells were completely unresponsive to IL-21. Naïve B cells from these individuals also failed to differentiate into memory cells *in vivo* (Figures 2 and 3). Thus, despite intact responsiveness to a suite of well-characterized B-cell growth and differentiation factors, the ability to receive signals through a γc-binding/JAK3-activating cytokine is a critical and rate-limiting step for the establishment of humoral immunity in humans (Figure 2) (121). Given the potency that IL-21 exerts on human B-cell differentiation, it was highly likely that this was the key γc-binding/JAK3-activating cytokine involved in human B-cell responses *in vivo*.

This was confirmed by the recent identification of individuals with homozygous loss-of-function mutations in *IL21R* that causes a novel PID, features of which include occasionally reduced serum IgG levels, poor Ab responses following vaccination with TD Ags (122), and a paucity of circulating memory B cells, including those expressing class switched Ig isotypes (107, 122). Not surprisingly, IL-21R-deficient naïve B cells exhibited impaired IL-21-induced proliferation, Ig class switching, and PC differentiation *in vitro*. This is consistent with a failure of IL-21 to mediate the acquisition of expression of *AICDA*, *PRDM1*, and *XBP1* in these cells, and mirrors the humoral immune defects observed in these patients. The cellular and molecular characterization of these patients has definitively established the criticality of IL-21 in establishing long-lived humoral immune responses. Furthermore, the finding that B cells with mutations in *IL2RG*, *JAK3*, or *STAT3* phenocopy IL-21R-deficient B cells, with respect to memory cell formation and responsiveness to IL-21, demonstrates that signaling downstream of the IL-21R/γc complex via JAK3 and STAT3 is essential for the effector function of IL-21 on B-cell differentiation in terms of generating efficient Ag-specific humoral immune responses (Figures 2 and 3). However, since serum levels of total IgM, IgG, and IgA are largely normal in most patients with mutations in either STAT3 or IL-21R, it is clear that the production of basal Ig is not dependent on IL-21R/STAT3 signaling. Indeed, as we have previously proposed (83), this is likely achieved by the interplay between ligands that do not signal via STAT3 – these could include many of the cytokines and factors detailed in this review (see Table 1), such as IL-4, IL-13, BAFF/APRIL as well as TLR ligands. Despite the availability of these ligands in STAT3- and IL-21R-deficient patients, and their ability to signal normally in IL-21R/STAT3-deficient B cells, these factors are collectively unable to compensate for impaired IL-21R signaling in order to generate a robust, long lasting Ag-specific Ab response.

Intriguingly, IL-21R-deficient individuals also have elevated levels of serum IgE (122), which is obviously also a feature of AD-HIES (113, 114, 116). Thus, it is likely that IL-21 also plays an important role in regulating IgE production by human B cells. However, whether this is due to a direct effect of IL-21 on B cells, or operates through an intermediate cell type [e.g., by inducing production of IFNγ by T cells and NK cells; (40, 100)] remains to be determined.

Lastly, it is worth commenting that prior to the discovery and subsequent characterization of IL-21, IL-10 was considered to be the most efficient cytokine capable of activating human B cells (31, 42, 48, 59). As IL-10 can also activate STAT3 (73), and STAT3-deficient human naïve B cells are unable to respond to the PC-inducing effects of IL-10 (83), it is possible that the humoral defects in AD-HIES patients reflects an inability to respond to not only IL-21 but also IL-10. However, since individuals with mutations in *IL-10* or *IL-10R* have intact specific Ab responses to vaccines (123), it is possible the IL-10 plays only a minor role in regulating human B-cell function *in vivo*. There are caveats to this conclusion, however, as most patients examined were young (<10 years old), and they also suffered from early onset inflammatory bowel disease (123). Thus, it remains plausible that IL-10 does contribute to B-cell function in healthy adults.

## IL-21/IL-21R and Systemic Autoimmune Diseases

Just as impaired signaling via IL-21 manifests as humoral immunodeficiency, aberrant or excessive IL-21-induced B-cell activation has been associated with the development of Ab-mediated autoimmune states in both murine models and human.

The first indication of a potential involvement of IL-21 in autoimmunity was the finding that IL-21 was overexpressed in several strains of mice (e.g., BXSB-*Yaa*, B6.Sle1-*Yaa*, Sanroque, MRL/MpJ-FAS^lpr/lpr^/J) that develop lupus-like disease (86, 124, 125). Furthermore, *in vivo* blockade of IL-21 ameliorated disease progression and severity in some of these settings (126–128), as well as in animal models of rheumatoid arthritis (129) and Sjogren’s syndrome (130). This was followed by the demonstration of elevated expression and/or production of IL-21 in human autoimmune conditions including SLE (131–134), rheumatoid arthritis (135), and Sjogren’s syndrome (136). Consistent with these findings, as well as with the recognition that IL-21 is predominantly produced by Tfh cells, it was perhaps not surprising that circulating Tfh-like cells have been detected in a broad array of autoimmune conditions including not only SLE, rheumatoid arthritis, and Sjogren’s syndrome, but also multiple sclerosis, autoimmune thyroid disease, myasthenia gravis, and juvenile dermatomyositis [reviewed in Ref. (6, 137)]. Importantly, the increases in Tfh cells generally correlated with numerous indices of disease severity, such as titers of autoAb, numbers of Ab-secreting plasmablasts, clinical scores, and even levels of serum IL-21. Furthermore, the expanded population of Tfh cells, as well as clinical features of each of these diseases, could be reduced following initiation and continuation of immunosuppressive treatments [reviewed in Ref. (6, 137)]. Independent confirmation that IL-21/IL-21R may be involved in the development of autoimmune diseases came from genome-wide association studies. Specifically, polymorphisms in either IL-21 or IL-21R genes have been identified that associated with SLE, RA, and primary Sjogren’s syndrome (138–141). Collectively, there is convincing evidence that IL-21 – most likely produced by Tfh cells – plays a pathological role in the initiation, development, and/or progression of several human autoimmune diseases caused by the production of autoantibodies.

## Concluding Comments and Future Perspectives

B cells play myriad fundamental roles in providing protective immunity against infection. However, the most prominent of these is the production of Ag-specific Ab following the terminal differentiation of B cells into long-lived PCs. This event is key to the establishment of long-term humoral immunity and memory, and underlies the success of most currently available vaccines. The criticality of Ab production by B cells to human health is evidenced by the pathological consequences of hypogammaglobulinemia, resulting in immunodeficiency. Conversely, the dysregulated production of excessive quantities of self-reactive Abs can be deleterious in the setting of autoimmunity. The detailed characterization of the effects of cytokines on B cells – from studies in genetically manipulated mice, *in vitro* cultures of human and murine B cells, and analysis of humans with specific PIDs – have revealed the central role that IL-21 has in generating memory B cells and specific Abs following exposure to TD Ags. Remarkably, alternative signals that could be integrated in B cells through other cytokine or co-stimulatory receptors are insufficient to initiate such B-cell responses when the IL-21/IL-21R signaling pathway is compromised. This paves the way for developing directed therapies to improve immune responses to vaccines or in immunocompromised individuals. Supporting this concept is the finding that administration of IL-21 to macaques increased frequencies of memory B cells as well as titers of virus-specific IgG (142). Conversely, therapies aimed at blocking the action of IL-21, either by directly targeting IL-21 itself or indirectly targeting Tfh cells or appropriate signaling molecules downstream of the IL-21R, so as to restrain the differentiation of rogue, autoreactive B cells into PCs, represents a feasible strategy for the treatment of various autoimmune diseases, as evidenced from numerous murine models (126–129, 143). Hopefully these findings will see successful translation to the clinic, thereby offering new hope for the treatment of these immune dyscrasias.

## Conflict of Interest Statement

The authors declare that the research was conducted in the absence of any commercial or financial relationships that could be construed as a potential conflict of interest.
